# Phenolic content discrimination in Thai holy basil using hyperspectral data analysis and machine learning techniques

**DOI:** 10.1371/journal.pone.0309132

**Published:** 2024-10-02

**Authors:** Apichat Suratanee, Panita Chutimanukul, Tanapon Saelao, Supachitra Chadchawan, Teerapong Buaboocha, Kitiporn Plaimas

**Affiliations:** 1 Department of Mathematics, Faculty of Applied Science, King Mongkut’s University of Technology North Bangkok, Bangkok, Thailand; 2 Intelligent and Nonlinear Dynamic Innovations Research Center, Science and Technology Research Institute, King Mongkut’s University of Technology North Bangkok, Bangkok, Thailand; 3 National Center for Genetic Engineering and Biotechnology (BIOTEC), National Science and Technology Development Agency, Klong Luang, Thailand; 4 Program in Bioinformatics and Computational Biology, Graduate School, Chulalongkorn University, Bangkok, Thailand; 5 Center of Excellence in Environment and Plant Physiology (CEEPP), Department of Botany, Faculty of Science, Chulalongkorn University, Bangkok, Thailand; 6 Omics Science and Bioinformatics Center, Faculty of Science, Chulalongkorn University, Bangkok, Thailand; 7 Center of Excellence in Molecular Crop, Department of Biochemistry, Faculty of Science, Chulalongkorn University, Bangkok, Thailand; 8 Advanced Virtual and Intelligent Computing (AVIC) Center, Department of Mathematics and Computer Science, Faculty of Science, Chulalongkorn University, Bangkok, Thailand; Universidade Federal do Para, BRAZIL

## Abstract

Hyperspectral imaging has emerged as a powerful tool for the non-destructive assessment of plant properties, including the quantification of phytochemical contents. Traditional methods for antioxidant analysis in holy basil (*Ocimum tenuiflorum* L.) are time-consuming, while hyperspectral imaging has the potential to rapidly observe holy basil. In this study, we employed hyperspectral imaging combined with machine learning techniques to determine the levels of total phenolic contents in Thai holy basil. Spectral data were acquired from 26 holy basil cultivars at different growth stages, and the total phenolic contents of the samples were measured. To extract the characteristics of the spectral data, we used 22 statistical features in both time and frequency domains. Relevant features were selected and combined with the corresponding total phenolic content values to develop a neural network model for classifying the phenolic content levels into ‘low’ and ‘normal-to-high’ categories. The neural network model demonstrated high performance, achieving an area under the receiver operating characteristic curve of 0.8113, highlighting its effectiveness in predicting phenolic content levels based on the spectral data. Comparative analysis with other machine learning techniques confirmed the superior performance of the neural network approach. Further investigation revealed that the model exhibited increased confidence in predicting the phenolic content levels of older holy basil samples. This study exhibits the potential of integrating hyperspectral imaging, feature extraction, and machine learning techniques for the rapid and non-destructive assessment of phenolic content levels in holy basil. The demonstrated effectiveness of this approach opens new possibilities for screening antioxidant properties in plants, facilitating efficient decision-making processes for researchers based on comprehensive spectral data.

## Introduction

*Ocimum tenuiflorum* L., commonly known as holy basil or tulsi, is a highly venerated herbaceous plant in the *Lamiaceae* family that flourishes in the warm, humid climates of tropical countries [1]. Holy Basil is renowned for its therapeutic properties, which can be attributed to its richness of phytochemical components including phenolics, flavonoids, phenylpropanoids, and terpenoids [2, 3]. Several studies have shown its potential anti-inflammatory activities [4–6], antioxidant activities [5, 7], and antimicrobial activities [8, 9]. Several of secondary metabolites in plants are phenolic compounds with antioxidant, anti-inflammatory, anti-aging, and other health-promoting properties [10, 11]. Therefore, phenolic content in Thai holy basil is explored and its antioxidant property has been investigated [12, 13].

Phenolic compounds represent a diverse class of phytochemicals found abundantly in holy basil. Among these, flavonoids such as orientin and vicenin, along with phenolic acids like rosmarinic acid, constitute significant portions of its chemical profile [14]. These compounds are renowned for their antioxidant properties, which play a crucial role in scavenging reactive oxygen species and counteracting oxidative stress-induced damage within the body [15]. Moreover, phenolics in holy basil demonstrate anti-inflammatory effects, potentially modulating various inflammatory pathways implicated in chronic diseases [15]. The utilization of phenolic content in holy basil presents promise for a range of health applications. From enhancing immune function to mitigating chronic inflammation and combating oxidative damage, the therapeutic potential of holy basil phenolics is extensive and multifaceted [15]. Additionally, emerging evidence suggests their role in modulating cellular signaling pathways associated with cancer progression, paving the way for novel strategies in cancer prevention and management [16].

Hyperspectral imaging has emerged as a powerful, non-invasive, and remote sensing tool for plant phenotyping, garnering significant attention in plant science research [17]. This advanced technique simultaneously captures both spectral and spatial information. In hyperspectral imaging, a light source illuminates the plant, and the reflected radiation is collected by objective lenses. The imaging spectrograph then splits or disperses the reflected light into different wavelengths, which are captured by hyperspectral sensors and converted into quantitative electrical signals, providing a wealth of data for analysis [18]. Hyperspectral imaging surpasses the limitations of the visible spectrum, capturing a vast array of spectral information that allows for an in-depth examination of plant characteristics and health status. By utilizing this innovative technology, researchers can gain a comprehensive understanding of plant physiology, vitality, and compositional attributes, opening new opportunities for scientific exploration and discovery. Hyperspectral imaging methods play crucial roles in the early detection and warning of plant diseases. Nguyen et al. (2021) utilized hyperspectral imaging with machine learning to detect the DNA virus grapevine vein-clearing virus (GVCV) at early asymptomatic stages [19]. They applied 2D and 3D convolutional layers to reflectance spectra signatures and extracted features for classification between healthy and GVCV-infected plants. Similarly, Nagasubramanian et al. (2019) employed a 3D deep convolutional neural network to learn hyperspectral data for identifying charcoal rot disease in soybean stems, observing differences between healthy and infected samples through reflectance spectra [20]. Moreover, hyperspectral imaging has shown potential for monitoring crop nutrient levels. De Silva et al. (2023) demonstrated the use of hyperspectral imaging for assessing nitrogen (N), phosphorus (P), potassium (K), calcium (Ca), copper (Cu), manganese (Mn), sulphur (S), and zinc (Zn) concentrations in macadamia leaves [21]. They extracted spectral data from images of both adaxial and abaxial leaf surfaces and used partial least squares regression (PLSR) models for analysis. In addition to disease detection and nutrient monitoring, Hyperspectral imaging has been applied to assess plant stress severity. Zhang et al. (2020) demonstrated the effectiveness of hyperspectral imaging in evaluating rice leaf blast severity. They calculated the spectral reflectance ratio (SRR) of rice leaves and employed support vector machine (SVM) models to assess blast severity across multiple growth stages [22]. Integration of hyperspectral imaging (HSI), neural networks (NN), and structural equation modeling has recently been performed by Mahmoodi-Eshkaftaki et al. [23] to determine feedstocks’ physicochemical characteristics and bio-H_2_ production. This research leverages advanced imaging techniques and machine learning models to extract meaningful chemical information from plant materials. The authors demonstrate that HSI can effectively capture spectral data across various wavelengths, which, when processed through NN, can yield accurate predictions of biomass properties. Principal component analysis was employed to identify important spectra used as input for the artificial neural network to predict feedstock characteristics and bio-H_2_ production. Additionally, structural equation modeling was used to evaluate the hypothetical response of bio-H_2_ production to the feedstock characteristics and the important spectra. Notably, their findings indicate the potential of specific spectral wavelengths in correlating with physicochemical characteristics, underscoring the relevance of wavelength selection in model training. This reinforces the applicability of HSI and NN in agricultural research, particularly in assessing phytochemical levels non-destructively, which is a critical advantage for studies focused on optimizing plant-based products. All of these studies collectively highlight the immense potential of hyperspectral imaging in various aspects of plant science research, paving the way for advanced plant phenotyping and precision agriculture.

In this study, we aimed to develop a non-destructive method for determining total phenolic content levels in holy basil (*Ocimum tenuiflorum* L.) using hyperspectral imaging combined with machine learning techniques that have been successfully applied in several applications [23–27]. Our objectives were to: (1) acquire and preprocess hyperspectral data from holy basil samples at various growth stages, (2) extract meaningful statistical features from the spectral data, and (3) develop and evaluate a machine learning model for classifying phenolic content levels. This novel approach addresses the limitations of traditional time-consuming antioxidant analysis methods and has the potential to revolutionize phytochemical assessment in medicinal plants. By enabling non-invasive assessment of phenolic content levels, our method could significantly impact quality control processes, enhance breeding programs, and contribute to more sustainable agricultural practices. Furthermore, this study serves as a proof-of-concept for the integration of hyperspectral imaging and machine learning in plant science, potentially paving the way for similar applications across various plant species and phytochemicals.

## Materials and methods

### Overview of analytical framework

**Fig 1:**
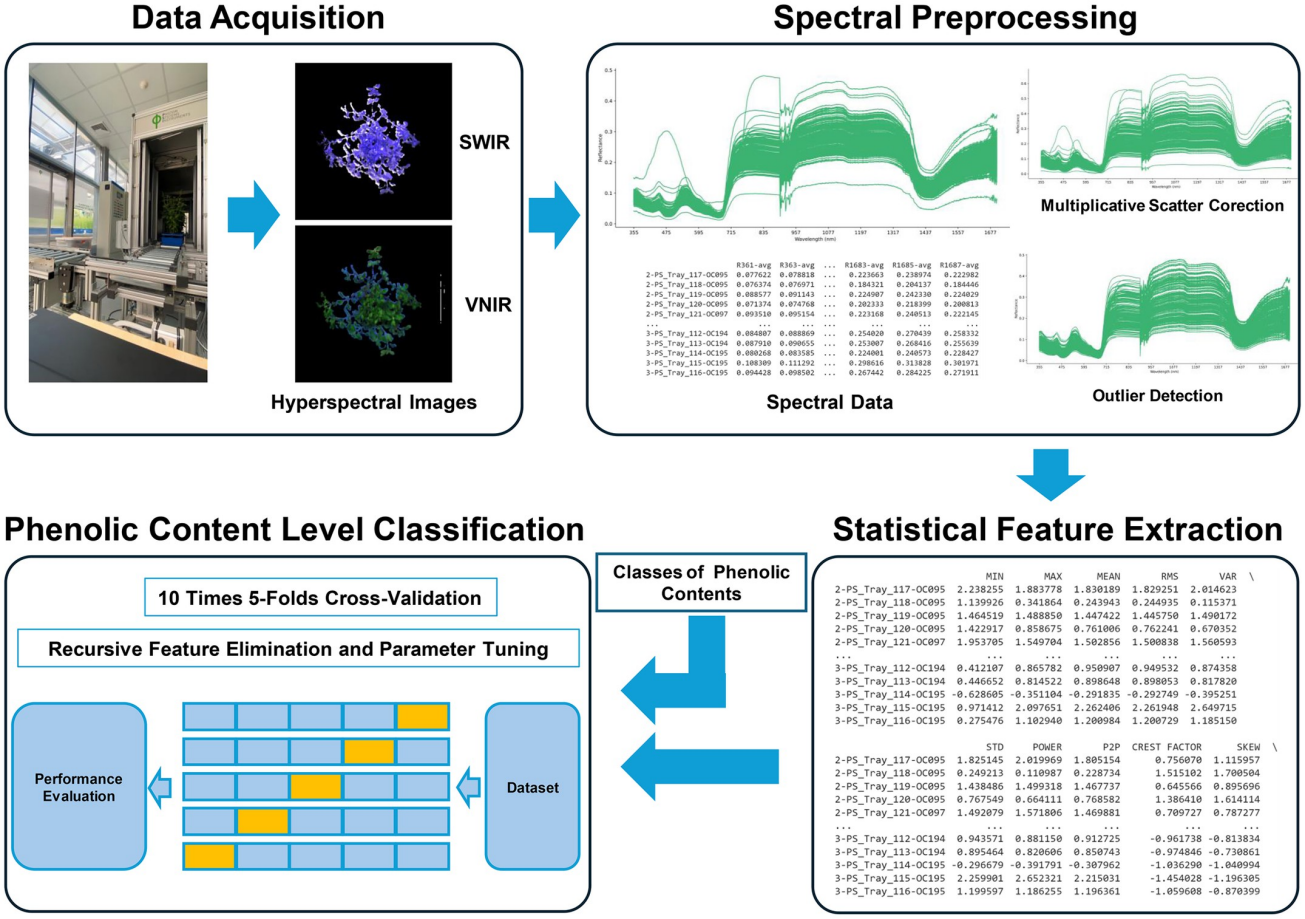
Schematic representation of the analytical framework employed in this study. This diagram provides an overview of the step-by-step approach and the key components involved in the analysis conducted as part of the present research work.

We investigated phenolic content in basil cultivars using hyperspectral imaging and machine learning. Hyperspectral images were collected for several basil cultivars. The raw spectral data underwent preprocessing to reduce light scattering effects and eliminate potential outliers. A comprehensive set of statistical features was then extracted from the preprocessed hyperspectral data for each cultivar. In parallel, laboratory analyses quantified the total phenolic content in each basil cultivar, providing reference values for the machine learning models. The extracted statistical features served as predictor variables for machine learning algorithms. These models were trained and optimized using the laboratory-determined phenolic levels as the target variable. We evaluated the predictive performance of the models using cross-validation techniques. Fig 1 presents a visual overview of this analytical framework.

### Sample description

#### Plant material and growth conditions

Twenty-six holy basil (*Ocimum tenuiforum* L.) accessions, encompassing both standard commercially available green (G) seeds from BENJAMITR ENTERPRISE (1991) CO., LTD. and red (R) seeds from Chia Tai Co. Ltd., Bangkok, Thailand. The Tropical Vegetable Research Center (TVRC) at Kasetsart University, Kamphaeng Saen Campus, Nakhon Pathom, Thailand, generously provided these accessions. Seed germination was conducted using the method outlined by Thongtip et al. [28] with slight modifications. All seeds were sown on a germination sponge (ESPEC Corp., Japan) under 100 μmol m⁻^2^ s⁻¹ photosynthetic photon flux density (PPFD) from white LEDs for 16-hour photoperiods each day. After 20 days, adhering to the conditions described by Chutimanukul et al. [13], the seedings were transplanted to a greenhouse. One-month-old plants were then transplanted into commercial peat moss substrate (Hortimed SIA, LATVIA) in plastic pots with a diameter of 20 cm. Each pot received 3 g of an inorganic 16-16-16 fertilizer (N-P-K; nitrogen, phosphorus from P₂O₅, potassium from K₂O). The plants were grown in a greenhouse at the Plant Phenomics Center, National Center for Genetic Engineering and Biotechnology (BIOTEC), National Science and Technology Development Agency (NSTDA), Pathum Thani, Thailand. The greenhouse environmental conditions included a 12-hour photoperiod with 250 μmol m⁻^2^ s⁻¹ PPFD, temperatures ranging from 28–32 °C, 75–90% relative humidity, and 400–800 μmol mol⁻¹ CO₂ concentration under natural sunlight.

Holy basil plants were harvested three times, each at full bloom: the first harvest occurred 42 days after transplanting, the second at 63 days after transplanting, and the third at 84 days after transplanting. Plant samples were imaged using a hyperspectral camera (Photon Systems Instruments, spol. s r.o., Czech Republic). These hyperspectral images were subsequently analyzed using PlantScreen^™^ data analysis software. The canopy leaves were collected for secondary phenolic quantification following hyperspectral imaging.

#### Total phenolic content

The extensive dataset used in this study was sourced from the National Center for Genetic Engineering and Biotechnology (BIOTEC), the National Science and Technology Development Agency (NSTDA). Fresh leaves of holy basil were harvested and dried in an oven at 40°C for 72 hours. Subsequently, the dried leaves were ground into a fine powder using mortar and pestle and stored at -20°C until further analysis. The plant extraction was conducted using a modified method [29]. Briefly, 10 mg of the powdered sample was added to 5 mL of absolute methanol solvent containing 1% HCl. The extracted solution was thoroughly mixed and incubated at 25 °C for 3 hours. After extraction, the solution was centrifuged at 12,000 rpm for 5 minutes utilizing an Eppendorf Centrifuge 5810R equipped with rotor F-34-6-38 (6x125g). The supernatant was transferred to a separate microcentrifuge tube (2 mL) for subsequent assessment of total phenolic content.

The total phenolic content (TPC) of the holy basil extract was determined using the modified Folin-Ciocalteu method with gallic acid as the standard [29, 30]. 200 μl of the extracted solution was combined with an equal volume of 1 N Folin-Ciocalteu reagent. Following a 15-minute incubation period at 25°C, 600 μl of 7.5% sodium carbonate (Na_2_CO_3_) solution was added to neutralize the mixture. The absorbance of the resulting solution was measured at 730 nm using a spectrophotometer (MultiskanSky, Thermo Scientific) after incubating for 1 hour at room temperature. TPC was determined using a standard gallic acid solution. The gallic acid solution was prepared and dissolved in water to establish calibration curves for calculating TPC concentrations. Results were expressed as milligrams of gallic acid equivalent (mg of GAE) per gram of sample dry weight.

#### Categorization criteria for phenolic content levels

The quantification of phenolic content levels was performed on both green and red basil cultivars using standardized methods. Our objective was to establish a threshold value that could effectively discriminate between samples with normal-to-high phenolic content levels and those with low phenolic content. To determine this threshold, we aimed for a slightly more flexible approach, rather than simply using the mean value, by incorporating the standard deviation. After calculating the average phenolic content across all samples from both cultivars, the threshold was set by subtracting one standard deviation from the mean value. Specifically, the average phenolic content was determined to be 28.8219 mg GAE/gDW, with a standard deviation of 5.4564 mg GAE/gDW. Consequently, the chosen threshold was set at 23.3655 mg GAE/gDW. This threshold value was subsequently utilized to categorize the samples into two groups: those with phenolic content levels below the threshold, considered as ’low,’ and those at or above the threshold, classified as ’normal-to-high.’ This categorization facilitated further analysis and enabled the evaluation of the predictive models’ performance in distinguishing between these two phenolic content level regimes.

### Hyperspectral data collection and preprocessing

#### Hyperspectral data collection

Hyperspectral image data of the fresh basil were acquired across the visible-near infrared (VNIR: 355–900 nm) and shortwave infrared (SWIR: 900–1700 nm) spectral ranges using a PlantScreen^™^ system. Each hyperspectral image comprises 510 x 500 pixels, yielding a total of 1116 bands within the range of 355 to 1700 nm. The hyperspectral images of holy basil are categorized into three groups based on their age, denoted as cuts: the first cut at 42 days, the second cut taken 21 days after the initial leaf cutting in the first cut, and the third cut aged 21 days after cutting the leaves of the second cut. To optimize the analysis and reduce the dimensionality of the dataset, we selected wavelengths at a regular interval of 2 nm from the spectral data obtained from the hyperspectral images. These hyperspectral images were subsequently analyzed using the PlantScreen^™^ data analysis software. This software converts the images and provides the spectral information statistics for each sample. This helps to reduce the number of variables and mitigate issues such as multicollinearity and overfitting. The images were first segmented to include only the holy basil parts, ignoring the background noise. Statistical information such as the average spectrum and standard deviation were computed by the PlantScreen^™^ software.

#### Data preprocessing and analysis

The spectral data from these three sets (or cuts) were analyzed to determine and compare the average values among groups by using a *t*-test. Significant differences were found in the data from the first and second cuts, as well as between the data from the first and third cuts. There was no significant difference found between the data from the second and third cuts (refer to the Results section for further explanation and discussion). Therefore, we selected to use only the spectral datasets from the second and third cuts for further analysis and machine learning. The raw hyperspectral data from the second and the third cuts underwent processing to remove artifacts and distortions caused by light scattering effects, employing a multiplicative scatter correction (MSC) technique [31, 32]. For the removal of outlier spectra, we calculated the z-score for each spectral record, and records with z-scores exceeding ±3.0 were considered outliers and subsequently eliminated.

### Feature extraction from the spectral data in time and frequency domain

With the filtered spectral data obtained, we proceeded to extract statistical features for each sample to characterize their properties. A total of 22 statistical features were calculated, comprising 14 time-domain features and 8 frequency-domain features. The time-domain features capture the signal’s temporal characteristics and reflect how the signal changes over time. These features included maximum value, minimum value, mean value, root mean square (RMS), variance, standard deviation, power, peak-to-peak amplitude, crest factor (the ratio of peak value to RMS), skewness (a measure of the signal’s asymmetry), kurtosis (a measure of the signal’s tailedness), form factor (the ratio of RMS to mean), pulse indicator (a measure of the signal’s pulsating nature), and margin (the difference between the maximum and minimum values). The extracted time-domain features are summarized in Table 1.

**Table 1:** Time-domain features calculated from the hyperspectral reflectance data, where *s*_*i*_ represents the reflectance value at wavelength *i* nm.

Feature	Calculation	
**Mean**	$S_{Mean}=\frac{1}{n}\sum_{i=1}^{n}s_{i}$	(1)
**Variance**	$S_{V}=\frac{\sum_{i=1}^{n}{\left(s_{i}-S_{Mean}\right)}^{2}}{n-1}$	(2)
**Standard deviation**	$S_{SD}=\sqrt{\frac{\sum_{i=1}^{n}{\left(s_{i}-S_{Mean}\right)}^{2}}{n-1}}$	(3)
**Maximum**	$S_{Max}=\text{max}(s_{i})$	(4)
**Minimum**	$S_{Min}=\text{min}(s_{i})$	(5)
**Peak-to-peak**	$S_{P2P}=\text{max}(s_{i})-\text{min}(s_{i})$	(6)
**Root mean square**	$S_{RMS}=\sqrt{\frac{1}{n}\sum_{i=1}^{n}{s_{i}}^{2}}$	(7)
**Power**	$S_{Power}=\frac{1}{n}\sum_{i=1}^{n}{s_{i}}^{2}$	(8)
**Crest factor**	$S_{CF}=\frac{\text{max}(s_{i})}{\sqrt{\frac{1}{n}\sum_{i=1}^{n}{s_{i}}^{2}}}$	(9)
**Skewness**	$S_{SK}=\frac{1}{n}\frac{\sum_{i=1}^{n}{\left(s_{i}-S_{Mean}\right)}^{3}}{{\left(S_{SD}\right)}^{3}}$	(10)
**Kurtosis**	$S_{KS}=\frac{1}{n}\frac{\sum_{i=1}^{n}{\left(s_{i}-S_{Mean}\right)}^{4}}{{\left(S_{SD}\right)}^{4}}$	(11)
**Form factor**	$S_{FF}=\frac{\sqrt{\frac{1}{n}\sum_{i=1}^{n}{s_{i}}^{2}}}{S_{Mean}}$	(12)
**Pulse indicator**	$S_{PI}=\frac{\text{max}(\left|s_{i}\right|)}{S_{Mean}}$	(13)
**Margin**	$S_{Margin}=\frac{\text{max}(\left|s_{i}\right|)}{{\left|\frac{1}{n}\sum_{i=1}^{n}\sqrt{\left|s_{i}\right|}\right|}^{2}}$	(14)

Furthermore, to observe the signal’s power distribution across different frequencies, we transformed the original signal using the fast Fourier transform (FFT). This transformation allowed us to analyze the signal in the frequency domain. From the transformed spectral data, we computed frequency-domain features such as the maximum value of the band power spectrum, the summation of band power spectrum values, the mean of band power spectrum, the variance of band power spectrum, the standard deviation of band power spectrum, the skewness of power spectral density, the kurtosis of power spectral density, and the relative spectral peak per band. These frequency-domain features provide insights into the signal’s frequency content and are summarized in Table 2.

**Table 2:** Frequency-domain features calculated from the hyperspectral reflectance data using the Fast Fourier Transform (FFT), where *f*_*i*_ represents the transformed value at frequency index *i* after applying the FFT.

Feature	Calculation	
**Mean of band power spectrum**	$F_{Mean}=\frac{1}{n}\sum_{i=1}^{n}f_{i}$	(15)
**Variance of band power spectrum**	$F_{V}=\frac{\sum_{i=1}^{n}{\left(f_{i}-F_{Mean}\right)}^{2}}{n-1}$	(16)
**Standard deviation of band power spectrum**	$F_{SD}=\sqrt{\frac{\sum_{i=1}^{n}{\left(f_{i}-F_{Mean}\right)}^{2}}{n-1}}$	(17)
**Maximum of band power spectrum**	$F_{Max}=\text{max}(f_{i})$	(18)
**Summation of band power spectrum**	$F_{Sum}=\sum_{i=1}^{n}f_{i}$	(19)
**Skewness of band power spectrum**	$F_{SK}=\frac{1}{n}\frac{\sum_{i=1}^{n}{\left(f_{i}-F_{Mean}\right)}^{3}}{{F_{V}}^{\frac{3}{2}}}$	(20)
**Kurtosis of band power spectrum**	$F_{KS}=\frac{1}{n}\frac{\sum_{i=1}^{n}{\left(f_{i}-F_{Mean}\right)}^{4}}{{F_{V}}^{\frac{4}{2}}}$	(21)
**Relative spectral peak per band**	$F_{RSP}=\frac{\text{max}(f_{i})}{\sqrt{\frac{1}{n}\sum_{i=1}^{n}f_{i}}}$	(22)

### Hyperparameters and architectures of machine learning algorithms

We employed several machine learning algorithms for classification, each with its own architecture and hyperparameters. The choice of these algorithms was based on their proven effectiveness in various classification tasks and their ability to handle complex, non-linear relationships in the data. For the neural network, we chose a feedforward architecture with three types of layers: an input layer, two hidden layers, and an output layer. This architecture was selected due to its ability to learn complex non-linear functions and its universal approximation capabilities. The ReLU activation function was used for the first and second hidden layers because it introduces non-linearity, is computationally efficient, and helps in alleviating the vanishing gradient problem [33]. The sigmoid function was chosen for the output layer to map the output to a probability between 0 and 1, which is suitable for binary classification tasks. The number of nodes in the hidden layer was considered a hyperparameter because it determines the complexity of the model. A larger number of nodes can capture more complex patterns but may lead to overfitting, while a smaller number may result in underfitting. We tuned this hyperparameter using a set of values (20, 40, 60) to find the optimal balance between model complexity and generalization. The number of epochs and batch size were also tuned as hyperparameters. The number of epochs determines the number of times the entire dataset is passed through the network during training, while the batch size specifies the number of samples used in each iteration of gradient descent. We used the sets (50, 100, 150) and (16, 32, 64) for epochs and batch size, respectively, based on common practices and the size of our dataset. To find the optimal hyperparameters, we employed the grid search technique using GridSearchCV from the scikit-learn package [34]. Grid search is a widely used method for hyperparameter tuning as it systematically evaluates all possible combinations of the specified hyperparameter values. We used three-fold cross-validation to ensure the robustness of the results and accuracy as the performance evaluation metric.

XGBoost is a gradient boosting framework that constructs an ensemble of decision trees. We chose XGBoost because it has been shown to outperform other tree-based algorithms in many classification tasks [35]. The hyperparameters we tuned for XGBoost include: the number of gradient boosted trees, the minimum sum of instance weight in a child, the minimum loss reduction required to make a further partition on a leaf node, the subsample ratio of the training instances, and the maximum depth of a tree. These hyperparameters control the complexity of the XGBoost model and its ability to capture patterns in the data while preventing overfitting. Random forest is an ensemble learning method that combines multiple decision trees to improve classification accuracy and reduce overfitting [36]. We chose random forest because it is robust to noise and can handle high-dimensional data effectively. The hyperparameters we tuned for random forest include the number of trees in the forest, the maximum depth of the trees, the minimum number of samples required to split an internal node, and the minimum number of samples required to be at a leaf node. These hyperparameters control the complexity of the individual trees and the overall ensemble, allowing us to find the optimal balance between model complexity and generalization. Bayesian classification is a probabilistic approach that uses Bayes’ theorem to make predictions [37]. We chose Bayesian classification because of its simplicity and effectiveness. For Bayesian classification, we used the GaussianNB classifier from the scikit-learn package and tuned the portion of the largest variance of all features that was added to variances for calculation stability. This hyperparameter helps in dealing with numerical instability and ensures the robustness of the classifier. The hyperparameter sets for each classification model are shown in S1 Table.

By tuning the hyperparameters of these algorithms, we aimed to find the optimal configuration that maximizes the classification accuracy on the given dataset while preventing overfitting. The choice of these algorithms and their hyperparameters was based on their theoretical properties, empirical performance, and suitability for the problem at hand.

### Model evaluation and feature selection

To evaluate the performance of the classification models and mitigate the risk of overfitting, we employed a rigorous ten times five-fold cross-validation technique with a balanced training dataset. In five-fold cross-validation, the dataset is randomly partitioned into five equally sized subsets. The model is then trained and evaluated five times, using each fold as the test set once while the remaining four folds serve as the training set. In each five-fold cross-validation run, every data point is used exactly once as part of the test set and four times as part of the training set. By repeating this process ten times with different random partitions, we ensure that each data point contributes to both model training and evaluation multiple times across the entire validation procedure. This comprehensive approach provides a more reliable estimate of the model’s performance across various data splits. In our study, we specifically used a ten times five-fold cross-validation approach, meaning that the five-fold cross-validation process was repeated ten times, with different random partitions of the data in each repetition. This strategy allows us to assess the model’s performance across multiple subsets of the data, reducing the likelihood of overfitting to any particular subset. By averaging the results across these ten repetitions, we obtained a more robust and stable assessment of the model’s performance, reducing the impact of random variations in the data partitioning. This technique provides a more reliable estimate of the model’s generalization capability, as it evaluates performance on data not seen during training. The consistency of results across these multiple iterations further supports the stability and reliability of our model, demonstrating its ability to perform well on unseen data. Through this process, we obtained prediction scores ranging from 0 to 1, and the overall approach helps in addressing concerns about overfitting while ensuring a thorough evaluation of the model’s performance. The overall performance was then assessed using a receiver operating characteristic (ROC) curve, illustrating the performance of a binary classification model at different decision thresholds. This curve plots the true positive rate (TPR) against the false positive rate (FPR). Lowering the classification threshold results in more items being classified as positive, leading to increases in both false positives and true positives. The area under the ROC curve, known as AUC, quantifies the entire two-dimensional area beneath the ROC curve. It indicates how effectively a model can generate relative scores to discriminate between positive or negative instances (‘normal-to-high’ or ‘low’ in our case) across all classification thresholds. The AUC score ranges from 0 to 1, with 0.5 representing random guessing and 1 indicating perfect performance. For accuracy calculation, the machine learning algorithms provided probability predictions for each instance, ranging from 0 to 1. To convert these probabilities into binary classifications for accuracy calculation, we applied a threshold of 0.5. Specifically, if the predicted probability was greater than 0.5, the instance was classified as 1 (’normal-to-high’), otherwise it was classified as 0 (’low’). The accuracy was then computed as the proportion of correct predictions (both true positives and true negatives) among the total number of cases examined. This metric provides a straightforward measure of the model’s overall correctness in classification. In addition, we employed an engineering metric, the a20-index [38, 39], to assess performance across different algorithms. The a20-index was originally used in engineering studies to calculate the percentage of samples with predicted values within ±20% deviation from the actual values, primarily for regression tasks. We adapted this concept for our classification task while maintaining the 20% threshold principle. For our binary classification with actual classes of 0 and 1, we counted samples with predicted probability values less than or equal to 0.2 for the actual class of 0, and samples with predicted probability values greater than or equal to 0.8 for the actual class of 1. The sum of these counts, termed m20, was divided by the total number of samples to calculate the a20-index. We computed the a20-index for all repetitions in our experiments for each algorithm and then calculated the average a20-index. The a20-index ranges from 0 to 1, with higher values indicating better performance.

To address the class imbalance issue, we applied undersampling techniques to the training set in each iteration of the procedure, creating a balanced dataset for training the classifier. The balanced training set was then used to train the model, while the test set was used to assess its performance on unseen data. This approach helped to mitigate the potential bias caused by class imbalance and ensured that the model’s performance was evaluated on a representative sample of the data.

To reduce the dimensionality of the feature space and eliminate irrelevant features, we incorporated the recursive feature elimination with cross-validation (RFECV) technique into the training process. The RFECV method employed logistic regression as an estimator and a three-fold cross-validation strategy to filter and select the most relevant features. By iteratively removing the least important features and assessing the model’s performance, RFECV identified the optimal subset of features that maximized the classification accuracy. These optimal features, obtained from the RFECV process, were then fed into machine learning algorithms to train and optimize the classification models.

This approach ensured that the models were built using only the most informative and discriminative features, thereby improving their predictive performance and generalization ability. The combination of undersampling techniques, RFECV feature selection, and cross-validation helped to mitigate the impact of class imbalance and irrelevant features, ultimately enhancing the robustness and reliability of the classification models. By employing a rigorous cross-validation strategy, we obtained a comprehensive and unbiased assessment of the models’ performance, providing confidence in their ability to generalize to new, unseen data.

## Results

### Spectral response across various growth stages

**Fig 2:**
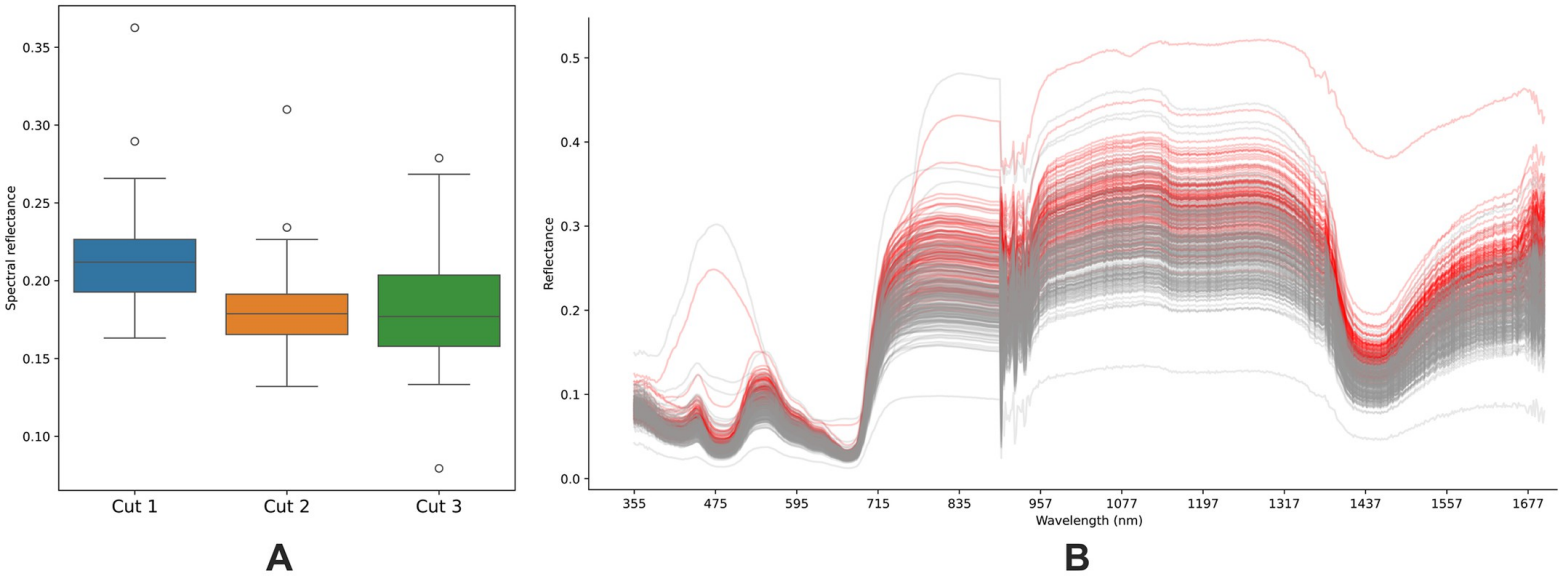
Spectral reflectance of holy basil samples. (A) The boxplot displays the distribution of average spectral reflectance values for each growth stage (Cut1, Cut2, or Cut3). (B) Raw spectral data of holy basil samples obtained through hyperspectral imaging. The red lines represent the spectral reflectance curves of the first cut (youngest) samples, while the grey lines correspond to the spectral reflectance curves of the second and third cuts (older samples) at different growth stages. The x-axis shows the wavelength range captured by the hyperspectral camera, and the y-axis indicates the relative reflectance intensity. The deviation of the first cut spectral reflectance patterns from the older samples can be observed, highlighting the differences in spectral response across various growth stages.

We obtained three sets of hyperspectral imaging data corresponding to different ages of holy basil. The spectral data from these three sets (or cuts) were analyzed to determine the average values within each group. Upon comparing the average values among the groups, significant differences emerged between the data from the first and second cuts, with a *p*-value of 2.7727e-21 obtained from the *t*-test. Similarly, a significant distinction was observed between the data from the first and third cuts, with a *p*-value of 1.8771e-15. However, there was no notable contrast found between the data from the second and third cuts (*p*-value of 0.7681). These findings suggest that the spectral dataset of the first cut deviates from that of the second and third cuts, as illustrated in the box plot presented in Fig 2A. This deviation could be attributed to the younger age of the holy basil plants in the first cut, resulting in higher reflectance compared to the older plants in the second and third cuts, as illustrated in Fig 2B. It is noteworthy that high reflectance can be indicative of low absorbance in younger age holy basils, given their inversely proportional relationship. Consequently, for further analysis, we selected to include only the spectral datasets from the second and third cuts to minimize the impact of age-related variations.

**Fig 3:**
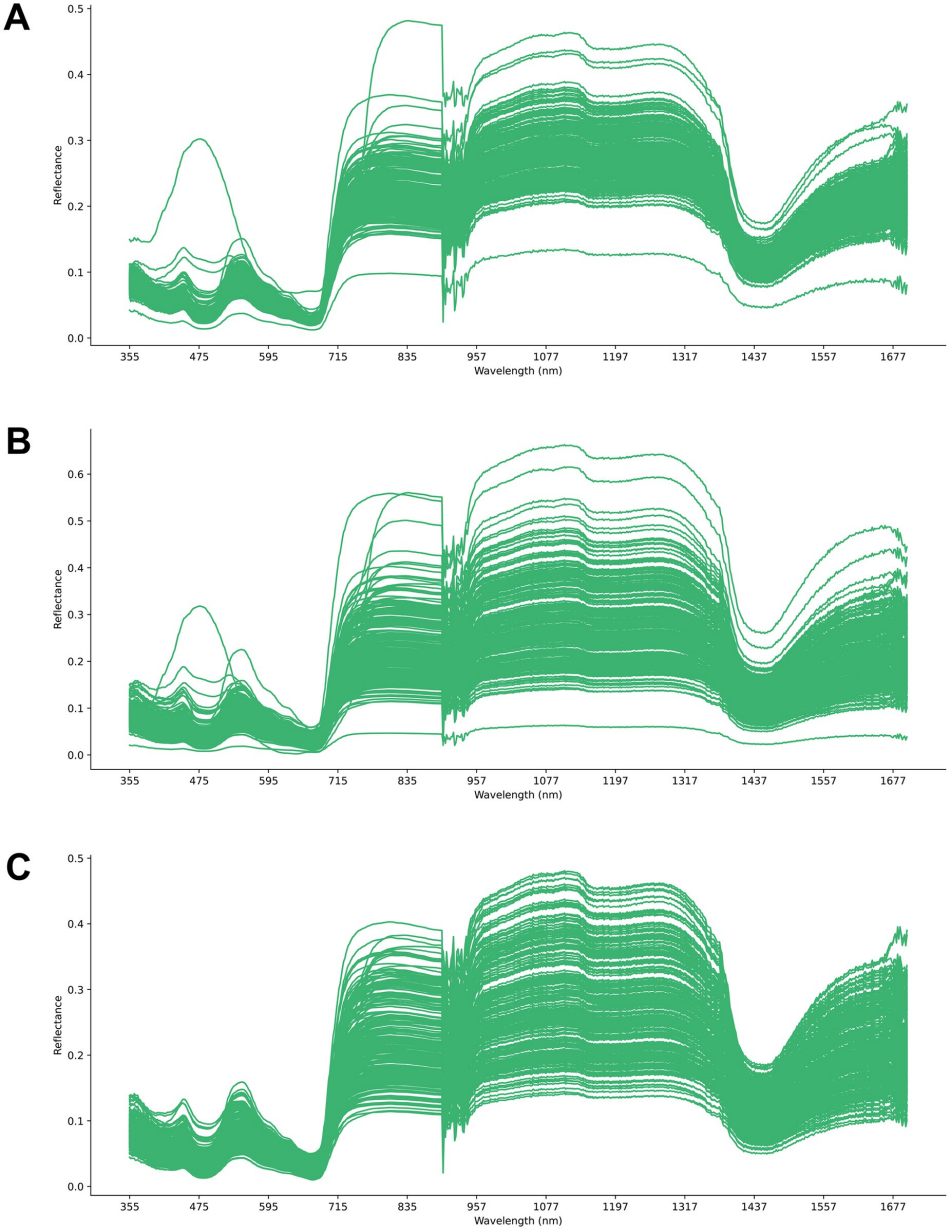
Preprocessing steps for the spectral data. (A) The original raw hyperspectral data. (B) The spectral data after applying multiplicative scatter correction to reduce multiplicative effects. (C) The corrected spectral data with outlier samples removed.

The raw hyperspectral data acquired from the 237 basil samples with different 26 cultivars aggregated from the second and the third cuts is displayed in Fig 3(A). This unprocessed data may contain artifacts and distortions caused by light scattering effects. To mitigate such issues, a multiplicative scatter correction (MSC) technique was employed. The result of this preprocessing step is illustrated in Fig 3(B), where the scattering-induced variations have been minimized. Following the MSC, outlier spectra were identified and removed using a robust statistical approach. Specifically, the z-score for each spectral record was calculated, and records with z-scores exceeding ±3.0 were considered outliers and subsequently eliminated. We removed 8 outlier samples from the spectral dataset. This outlier removal process ensured the exclusion of potentially erroneous or anomalous data points. Fig 3(C) presents the final preprocessed hyperspectral data, free from scattering artifacts and outliers, ready for further analysis and feature extraction.

### Distributions of statistical spectral features

Our dataset consisted of 229 basil samples, each characterized by a total of 22 statistical features extracted from both the time and frequency domains. Following the predetermined categorization criteria based on phenolic content levels, we classified the samples into two groups: 138 samples were categorized as having normal-to-high phenolic content, while the remaining 91 samples were classified as having low phenolic content. Prior to proceeding with the machine learning step, we performed feature normalization to transform the extracted features into a standardized range of values, ensuring consistent scaling across all features.

**Fig 4:**
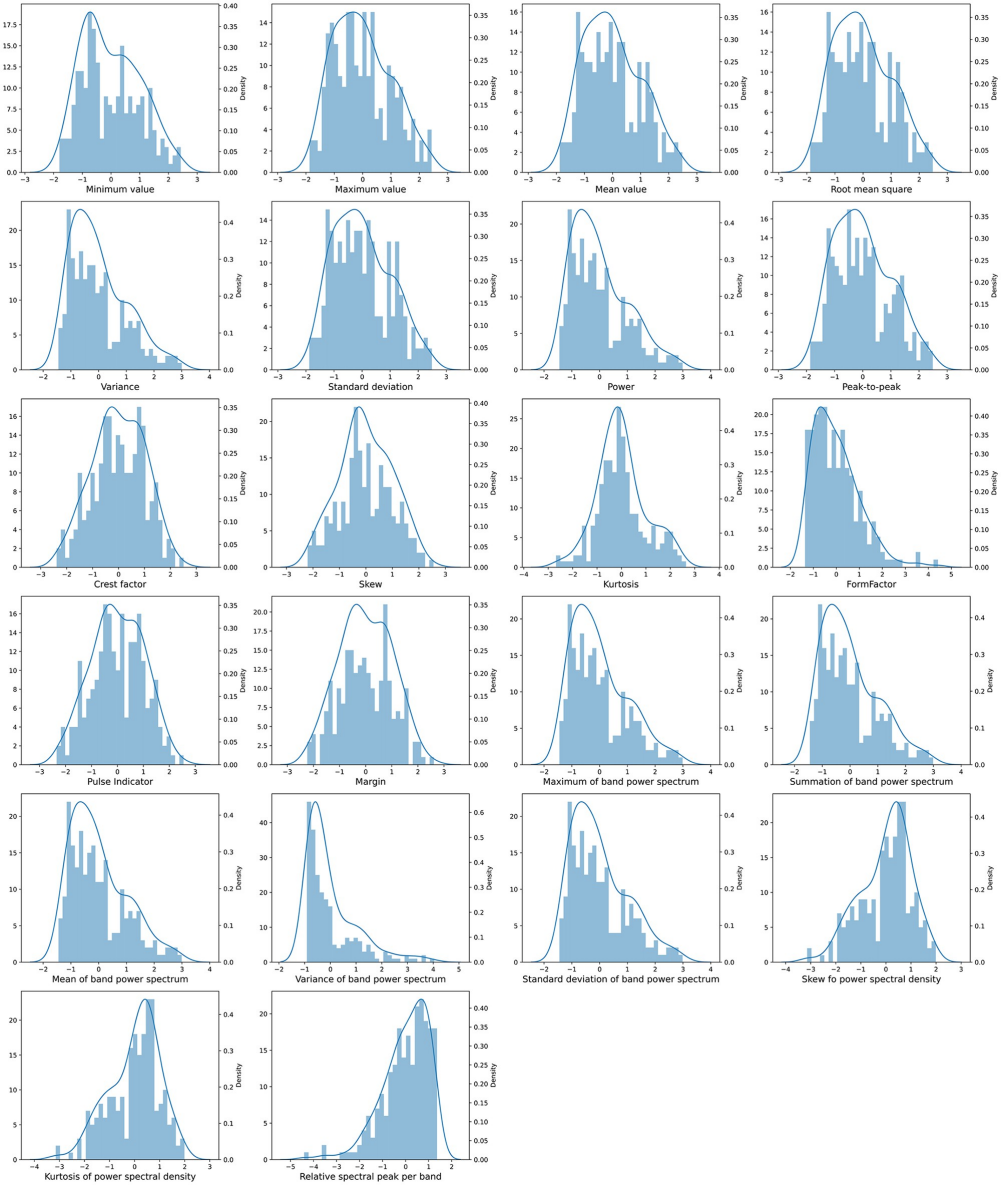
Distributions of the 22 statistical features extracted from the time-domain and frequency-domain spectral data of 229 basil samples. The features were derived from filtered spectral data and included 14 time-domain features such as maximum, minimum, mean, variance, and skewness, and 8 frequency-domain features such as band power spectrum characteristics and power spectral density moments.

Upon examining the distributions of the extracted features, we noticed similarities between certain pairs of features, indicating potential redundancy or multicollinearity. For instance, the mean and root mean squared (RMS) features exhibited highly correlated values, as did the maximum band power and summation of band power spectrum features. The distributions of features are shown in Fig 4. Such similarities among features can potentially introduce noise and adversely affect the performance of machine learning models. Consequently, we recognized the necessity of incorporating a feature selection process during the training phase to identify and retain the most informative and non-redundant subset of features.

### Optimal spectral features for basil phenolic level classification

During the ten times five-fold cross-validation training process, the RFECV analysis identified a set of optimal features for each iteration. These optimal features were collected and analyzed to determine their frequency of use and importance in the model’s performance. On average, 13 features were selected per iteration. Notably, the top 8 features belonged to the time-domain feature group, with skewness and kurtosis emerging as the most important features, each appearing 50 times in the top features list. The equal frequency of these two features highlights their significance and suggests an equal contribution to the model’s performance. Crest factor was the second most significant feature, appearing 49 times, closely followed by pulse indicator, the third most prevalent feature, which appeared 48 times. These findings emphasize the crucial role of time-domain features in the analysis.

Among the frequency-domain features, the skewness and kurtosis of the band power spectrum were the two most frequently used, mirroring the top features in the time-domain group. The skewness of the band power spectrum was used more frequently, appearing 37 times, while the kurtosis of the band power spectrum appeared 33 times. Although the top features were from the time-domain group, the high frequency of use of these frequency-domain features underscores their importance in the analysis. The prominence of skewness and kurtosis in both time-domain and frequency-domain groups further highlights their significance as key features in this study. S2 Table provides a summary of the features and their frequency of use, presenting the most important time-domain and frequency-domain features that contributed to the model’s performance.

### Efficacies of classification algorithms

To evaluate the performance of our approach, we conducted experiments using several alternative classification algorithms, including XGBoost (Extreme Gradient Boosting), random forest, and naïve Bayes. By employing the same data and pre-processing steps, we aimed to provide a fair comparison between these methods and our neural network classifiers. The results demonstrated that the neural network classifiers outperformed the other algorithms in terms of both AUC and accuracy. XGBoost emerged as the second-best performing algorithm, achieving an AUC of 0.8017 and an average accuracy of 0.7301, which closely followed the neural network results. Random forest classifiers also exhibited good performance, with an AUC of 0.7843 and an average accuracy of 0.7297, although slightly lower than XGBoost. On the other hand, the naïve Bayes classifier had the lowest performance among the compared algorithms, with an average AUC of 0.6625 and an average accuracy of 0.6511, indicating that it may not be the most suitable choice for this specific classification task. In addition, we assessed the performances of these four algorithms using the a20-index and found that the results corresponded to the AUCs and accuracies. The neural network showed the highest index value of 0.7485. XGBoost and random forest showed index values of 0.7275 and 0.7114, respectively. The naïve Bayes classifier yielded the lowest index value of 0.6856. These findings highlight the superior efficacy of the neural network and XGBoost classifiers in accurately predicting the phenolic content levels, and further validating the effectiveness of our proposed approach. The summarized performances of these algorithms are shown in Table 3, and the complete list of performance metrics is shown in S3 Table.

**Table 3:** Performance of different machine learning algorithms.

Algorithms	The average of the area under the curve (AUC) values	The average of the accuracy values
**Neural network**	0.8113	0.7345
**Extreme Gradient Boosting (XGBoost)**	0.8017	0.7301
**Random forest**	0.7843	0.7297
**Bayes classification**	0.6625	0.6511

**Fig 5:**
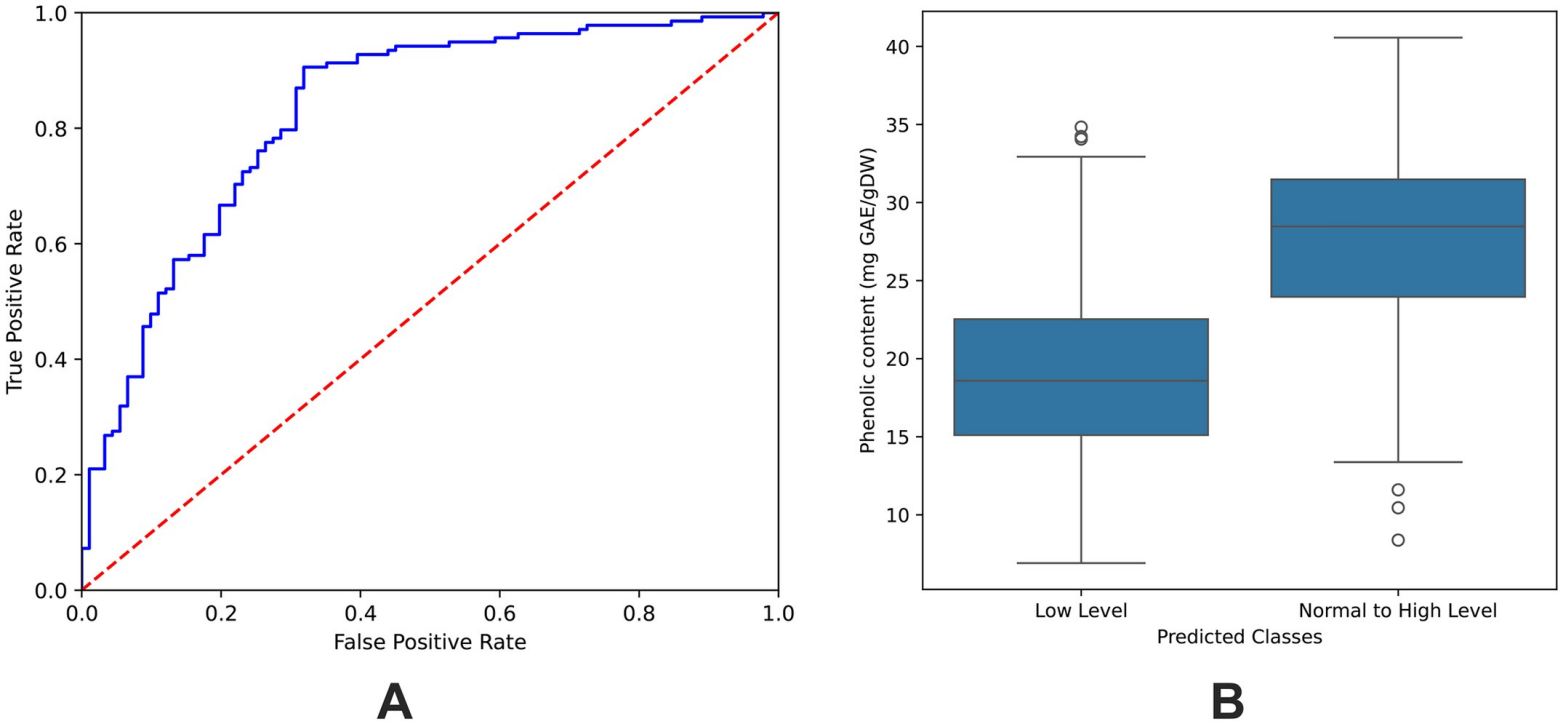
Classification performance and discrimination of phenolic contents using hyperspectral data. (A) Receiver Operating Characteristic (ROC) curve illustrating the performance calculated based on the predicted scores from machine learning. (B) Box plot illustrating the discrimination of actual phenolic contents into low and normal-to-high levels based on predicted scores.

Therefore, we utilized a neural network algorithm for the phenolic content classification. To ensure reliable and generalizable results, we adopted a ten-times five-fold cross-validation strategy, where the dataset was repeatedly partitioned into training and testing subsets, and the model’s performance was evaluated across multiple rounds. The ROC curve and the discrimination in predicted phenolic contents are shown in Fig 5. The performance results of this machine learning demonstrate the effectiveness of our feature extraction, selection, and machine learning pipeline in classifying basil samples into different phenolic content levels using the hyperspectral image data.

We used the predicted probabilities from machine learning to discriminate between samples with low phenolic content and those with normal-to-high phenolic content. Each sample was assigned ten predicted probability values based on ten repetitions of five-fold cross-validation. We then computed the median of these predicted probability values and assigned it as a score for each sample. To observe the discrimination in the samples using the prediction scores, we investigated the ROC curve that shows the trade-off between sensitivity (TPR) and specificity (1-FPR) as the classification threshold is varied (see Fig 5(A)). We attempted to find an optimal threshold that strikes a balance between maximizing true positives while minimizing false positives. With this, we defined the optimal threshold by maximizing the Youden’s J statistic, which represents the best balance between sensitivity and specificity. We obtained the optimal threshold of 0.3366. A sample that has a score greater than or equal to this threshold was assigned to the normal-to-high phenolic content level; otherwise, it was assigned to the low phenolic content level. Fig 5(B) shows the box plot discriminating between low and normal-to-high levels based on the predicted score. These two groups were significantly different with a *p*-value of 1.5258e-21 using a statistical *t*-test. This shows that the score can be used to discriminate between these two groups.

### Determining high phenolic content samples from machine learning predictions

We investigated samples with high confidence of having normal-to-high phenolic content. It is important to note that these predicted samples were chosen based on the predicted score from machine learning with hyperspectral data, indicating that they have normal-to-high phenolic content. To further increase the confidence in our results, we filtered the data to include only samples with phenolic content confirmed to be in the normal-to-high range based on laboratory analysis. When we selected only the sample with a prediction score greater than or equal to 0.95, we obtained 21 samples, of which 17 were from third cut. When we lowered the threshold to include samples with predicted samples greater than or equal to 0.9, we obtained 38 samples, with 32 of these being from the third cut. For the predicted samples with scores greater than or equal to 0.8, this process yielded a total of 58 samples. We found that 47 out of the 58 samples were from the third cut, while only 11 samples were from the second cut. This finding suggests that older holy basil plants, as represented by the third cuts, tend to have higher phenolic content compared to younger plants, as represented by the second cuts. The combination of predictions from machine learning with hyperspectral data and laboratory-confirmed phenolic content strongly supports this conclusion. The list of high-confidence samples with scores greater than or equal to 0.95 is shown in Table 4. The complete list of samples with predicted scores is in S4 Table. S1 Fig presents a stacked bar plot showing the varying numbers of predicted samples from cuts 2 and 3 for different score thresholds. The plot highlights the distinct sample counts between the two cuts in the predicted samples.

**Table 4:** High phenolic content samples with scores greater than or equal to 0.95 and phenolic content values belonging to the group of normal-to-high phenolic content.

Accession ID	Score	Phenolic content (mg GAE/gDW)	Cut	Code	Location
3-PS_Tray_090-OC063	0.9956	33.1466	3	OC063	Chai Badan, Lopburi
3-PS_Tray_111-OC194	0.9931	30.1220	3	OC194	Udon Thani
3-PS_Tray_094-OC064	0.9926	31.6475	3	OC064	Roi Et
3-PS_Tray_082-OC057	0.9904	30.0168	3	OC057	Damnoen saduak, Ratchaburi
3-PS_Tray_200-Green	0.9896	36.5657	3	Green	Chia Tai Co. Ltd., Bangkok, Thailand
3-PS_Tray_097-OC072	0.9884	28.4914	3	OC072	Wat Phleng, Ratchaburi
3-PS_Tray_108-OC135	0.9880	29.1489	3	OC135	Mueang Phitsanulok, Phitsanulok
3-PS_Tray_115-OC195	0.9879	26.4926	3	OC195	Kumphawapi, Udon thani
3-PS_Tray_091-OC063	0.9874	29.9905	3	OC063	Chai Badan, Lopburi
3-PS_Tray_193-OC148	0.9861	34.3301	3	OC148	Mae Lao, Chiang Rai
3-PS_Tray_107-OC113	0.9771	31.2793	3	OC113	Mueang Rayong, Rayong
3-PS_Tray_194-OC148	0.9761	30.2725	3	OC148	Mae Lao, Chiang Rai
2-PS_Tray_211-Green	0.9723	26.1395	2	Green	Chia Tai Co. Ltd., Bangkok, Thailand
2-PS_Tray_208-Green	0.9717	24.1623	2	Green	Chia Tai Co. Ltd., Bangkok, Thailand
3-PS_Tray_180-OC141	0.9675	27.5972	3	OC141	Mueang Phrae, Phrae
2-PS_Tray_146-OC130	0.9661	25.9928	2	OC130	Mueang Lampang, Lampang
2-PS_Tray_175-OC141	0.9562	27.8602	2	OC141	Mueang Phrae, Phrae
3-PS_Tray_104-OC081	0.9540	33.5937	3	OC081	Bang Klam, Songkhla
3-PS_Tray_196-OC148	0.9536	30.5954	3	OC148	Mae Lao, Chiang Rai
3-PS_Tray_113-OC194	0.9500	30.1220	3	OC194	Udon Thani

## Discussion

In this study, the obtained hyperspectral imaging data provided valuable insights into the spectral characteristics of holy basil plants at different ages. Our analysis revealed significant differences in spectral profiles between the age groups, particularly between the first cut (representing younger plants) and the second and third cuts (representing older plants). The observed discrepancies in spectral data suggest age-related variations in the biochemical composition and physiological properties of holy basil plants. Specifically, the higher reflectance detected in the first cut aligns with previous studies indicating lower absorbance in younger plants due to differences in chlorophyll content, leaf structure, and developmental stage. These differences in spectral signatures likely stem from variations in pigments, leaf thickness, and cellular structures, which influence light absorption and reflection properties. The absence of significant differences between the second and third cuts suggests a certain level of spectral stability or uniformity in older holy basil plants. This consistency may reflect the maturation process reaching a relatively steady state in terms of biochemical composition and physiological responses.

Interestingly, a recent study by Mahmoodi-Eshkaftaki et al. [23] analyzed hyperspectral imaging data using principal component analysis (PCA) and neural networks to predict biomass characteristics of feedstock. They found a close relationship between spectral reflectance and physicochemical characteristics of the feedstock. Consequently, they used spectra to estimate the physicochemical characteristics of samples obtained from preprocessed tomato waste. The aspect most relevant to our study is their method of determining the most significant spectra using PCA. For instance, they identified that wavelengths between 500–650 nm have high PCA coefficients and are important for determining feedstock characteristics such as tannin, chlorophyll, and carbohydrate contents. This finding is particularly interesting for our future studies, as it suggests a potential approach for determining significant spectra to identify phenolic content in Thai holy basil.

Through employing diverse machine learning methodologies, our results demonstrate that the neural network algorithm achieved the best performance among the compared models. However, it is important to note that this high performance comes at the cost of increased computational time compared to other algorithms. While the XGBoost algorithm yielded slightly lower AUC and accuracy scores, its computational efficiency was significantly better than that of the neural network, being 3 times faster. XGBoost was able to train and make predictions much faster, making it a more practical choice in scenarios where computational resources or time is limited. The choice between the neural network and XGBoost algorithms ultimately depends on the specific requirements and constraints of the study. If the highest possible performance is the primary goal and computational resources are readily available, the neural network algorithm may be the preferred choice. However, if the study requires faster training and prediction times, or if computational resources are scarce, XGBoost provides a good balance between performance and efficiency.

Both algorithms require a careful design of their architectures, which affects the number of parameters. The high number of parameters allows neural networks to learn complex patterns and relationships in the data, contributing to their superior performance. However, this also means that neural networks require more computational resources and time to train and optimize these parameters. Neural networks have a large number of parameters with multiple hidden layers. The hyperparameters, including the number of units in each layer, batch size, and epochs, also affect the performance. Therefore, tuning parameter processes are crucial. On the other hand, XGBoost typically has fewer parameters to tune compared to neural networks. The main parameters in XGBoost include the number of trees, maximum depth of the trees, learning rate, and regularization parameters. While XGBoost still requires careful tuning of these parameters to achieve optimal performance, the search space is generally smaller compared to neural networks. This can make the parameter tuning process more manageable and less time-consuming. The reduced number of parameters in XGBoost also contributes to its faster training and prediction times. With fewer parameters to optimize, XGBoost can converge more quickly and make predictions more efficiently. This can be particularly advantageous in scenarios where the model needs to be frequently updated or deployed in real-time applications. The architecture and design of the neural network, as well as the quality and relevance of the input features, also play crucial roles in its performance. Similarly, the effectiveness of XGBoost depends on the careful selection of features and the appropriate tuning of its parameters. In conclusion, while the neural network algorithm achieved the highest performance in our experiments, it comes with the trade-off of increased computational time and a larger number of parameters to tune. XGBoost, with its fewer parameters and faster training and prediction times, offers a more computationally efficient alternative, albeit with slightly lower performance. The choice between these algorithms should be based on the specific requirements of the project, considering factors such as performance targets, available computational resources, and the need for efficient model training and deployment.

By utilizing machine learning algorithms to analyze hyperspectral images, our study aims to develop a non-invasive and rapid method for detecting antioxidant content in holy basil. This approach eliminates the need for time-consuming and resource-intensive wet lab experiments, streamlining the process of assessing the antioxidant properties of basil samples. The development of a tool capable of accurately detecting antioxidant content in holy basil can greatly benefit agricultural planning processes. Farmers and agricultural professionals can use this tool to assess the quality and nutritional value of basil crops quickly and efficiently. By obtaining real-time information on phenolic contents, farmers can make informed decisions regarding harvesting schedules and optimize crop management practices to ensure the production of high-quality basil products. The ability to classify holy basil based on phenolic contents using hyperspectral imaging enables enhanced quality control measures in the agricultural industry. By accurately assessing antioxidant levels, producers can ensure the consistency and standardization of basil products, meeting regulatory requirements and consumer expectations for quality and nutritional value.

## Conclusion

In this study, we successfully analyzed the spectral data obtained from hyperspectral imaging of 26 Thai holy basil (*Ocimum tenuiflorum* L.) cultivars at three different growth stages. Total phenolic contents of the samples were measured in parallel. The spectral data underwent preprocessing and feature extraction in both time and frequency domains, yielding 22 statistical features. Relevant features were selected for further analysis. By combining the optimal statistical features from the spectral data with the corresponding total phenolic content levels, we successfully developed a neural network model to infer the phenolic content levels (low and normal-to-high levels) based on the spectral data. The model achieved an AUC of 0.8113 and an accuracy of 0.7346, demonstrating its effectiveness in classifying phenolic content levels in holy basil. To evaluate the performance of the established neural network model, we compared it with other machine learning models for classification. The neural network model exhibited superior performance, highlighting its suitability for this task. Further investigation of the predicted results revealed that the model exhibited higher confidence in predicting the phenolic content levels of older holy basil samples. Based on the machine learning results and data analysis, samples classified as ’normal-to-high’ with high prediction scores were predominantly from the third cut. This observation supports the conclusion that holy basil plants exhibit significant age-related differences in their spectral characteristics. These differences likely reflect changes in biochemical composition, leaf structure, and physiological properties as the plants mature.

This study demonstrates the innovative integration of hyperspectral imaging, advanced feature extraction techniques, and machine learning for the rapid and non-destructive assessment of phenolic content levels in holy basil. Our novel approach, combining time and frequency domain features with neural network modeling, offers a significant advancement in phytochemical analysis. The superior performance of our neural network model, compared to traditional methods and other machine learning techniques, underscores the potential of this approach to revolutionize quality control processes in plant production. Furthermore, the observed increase in model confidence with plant age provides unique insights into the dynamics of phenolic content accumulation, opening new avenues for optimizing harvest times and breeding strategies. Future research could focus on expanding the sample size, exploring additional spectral features, and validating the model’s performance across different environmental conditions and cultivation practices. This would further enhance the robustness and applicability of our approach in real-world scenarios, potentially extending its use to other medicinal plants and phytochemicals, thus contributing to broader advancements in plant science and agriculture.

## Supporting information

S1 Table List of hyperparameters for classification models. (DOCX)

S2 Table Top statistical features identified by the recursive feature elimination with cross-validation (RFECV) analysis and their frequency of use. (DOCX)

S3 Table The average performances of different machine learning algorithms. (PDF)

S4 Table The complete list of samples with predicted scores. (XLSX)

S1 Fig The stacked bar plot illustrates the different number of predicted samples from cut 2 and cut 3 for various score thresholds. (TIF)

## Abbreviations

AUC: the area under the curve
CF: Crest factor
CV: Cross-validation
FF: Form factor
FFT: the fast Fourer transform
FPR: the false positive rate
GAE: Gallic Acid Equivalent
gDW: grams of Dry Weight
GVCV: grapevine vein-clearing virus
HSI: Hyperspectral Imaging
KS: Kurtosis
Max: Maximum
mg: Milligrams
Min: Minimum
MSC: a multiplicative scatter correction
NN: Neural network
P2P: Peak-to-peak
PI: Pulse indicator
PPFD: Photosynthetic photon flux density
RFECV: the recursive feature elimination with cross-validation
RMS: root mean square
ROC: a receiver operating characteristic
RSP: Relative spectral peak per band
SD: Standard deviation
SK: Skewness
SRR: Spectral reflectance ratio
SWIR: shortwave infrared
TPR: the true positive rate
V: Variance
VNIR: visible-near infrared
XGBoost: Extreme Gradient Boosting
μmol: Micromoles
