# Supplementary material for: Phenolic content discrimination in Thai holy basil using hyperspectral data analysis and machine learning techniques
Source: PLoS One. 2024 Oct 2;19(10):e0309132. doi: 10.1371/journal.pone.0309132 (PMC11446419; doi:10.1371/journal.pone.0309132)
Supplement: S1 Table — (DOCX) [file pone.0309132.s001.docx]

**S1 Table.** List of hyperparameters for classification models

| **Model** | **Function (Package)** | **Hyperparameter values for grid search algorithm** |
| --- | --- | --- |
| Neural Network | KerasClassifier (Scikit-Learn Wrapper for Keras): Relu, Relu, and Sigmoid activation functions for layers | unit = (20, 40, 60)  batch_size = (16, 32, 64)  epoch = (50, 100, 150) |
| XGBoost | XGBClassifier (xgboost) | booster = gbtree tree_method = gpu_hist objective = (binary:logistic) n_estimators = (4, 6, 9, 12, 20) min_child_weight = (0.2, 0.4, 0.6, 0.8, 1.0) gamma = (1, 3) subsample = (0.1, 0.4, 1.0) colsample_bytree = 1.0 max_depth = (2, 3, 5, 10) |
| Random Forest | RandomForestClassifier (Scikit-learn) | n_estimators = (4, 6, 9, 12, 20) max_features = sqrt criterion = gini max_depth = (3, 5, 15, 20) min_samples_split = (2, 3, 5) min_samples_leaf = (5, 8, 12) |
| Naïve Bayes Classifier | GaussianNB (Scikit-learn) | var_smoothing = (1e-12, 1e-9, 1e-6, 1e-3) |
