# Supplementary material for: Phenolic content discrimination in Thai holy basil using hyperspectral data analysis and machine learning techniques
Source: PLoS One. 2024 Oct 2;19(10):e0309132. doi: 10.1371/journal.pone.0309132 (PMC11446419; doi:10.1371/journal.pone.0309132)
Supplement: S2 Table — (DOCX) [file pone.0309132.s002.docx]

**S2 Table.** Top statistical features identified by the Recursive Feature Elimination with Cross-validation (RFECV) analysis and their frequency of use.

| **Statistical feature** | **Frequency of use** |
| --- | --- |
| Skewness | 50 |
| Kurtosis | 50 |
| Crest factor | 49 |
| Pulse indicator | 48 |
| Maximum | 44 |
| Peak-to-peak | 42 |
| Minimum | 41 |
| Margin | 39 |
| Skewness of band power spectrum | 37 |
| Kurtosis of band power spectrum | 33 |
| Variance of band power spectrum | 33 |
| Mean | 31 |
| Root mean square | 28 |
| Standard deviation | 27 |
| Relative spectral peak per band | 23 |
| Form factor | 20 |
| Standard deviation of band power spectrum | 18 |
| Variance | 15 |
| Mean of band power spectrum | 11 |
| Maximum of band power spectrum | 11 |
| Summation of band power spectrum | 9 |
| Power | 9 |
