# Supplementary material for: Phenolic content discrimination in Thai holy basil using hyperspectral data analysis and machine learning techniques
Source: PLoS One. 2024 Oct 2;19(10):e0309132. doi: 10.1371/journal.pone.0309132 (PMC11446419; doi:10.1371/journal.pone.0309132)
Supplement: S3 Table — (PDF) [file pone.0309132.s003.pdf]

S3 Table: The average performances of different machine learning algorithms.

Algorithm	The average of the area under the curve (AUC) values	The average of accuracy values	The average of precision values	The average of sensitivity values	The average of specificity values	The aveage of true positive values	The average of false negative values	The average of false positive values	The average of true negative values
Neural network	0.8113	0.7345	0.8035	0.7406	0.7253	20.44	7.16	5.00	13.20
Extreme gradient boosting (XGBoost)	0.8017	0.7301	0.8005	0.7355	0.7219	20.30	7.30	5.06	13.14
Random Forest	0.7843	0.7297	0.7788	0.7703	0.6681	21.26	6.34	6.04	12.16
Bayes classification	0.6625	0.6511	0.6972	0.7442	0.5099	20.54	7.06	8.92	9.28
