# Supplementary figures and images for: Phenolic content discrimination in Thai holy basil using hyperspectral data analysis and machine learning techniques
Source: PLoS One. 2024 Oct 2;19(10):e0309132. doi: 10.1371/journal.pone.0309132 (PMC11446419; doi:10.1371/journal.pone.0309132)

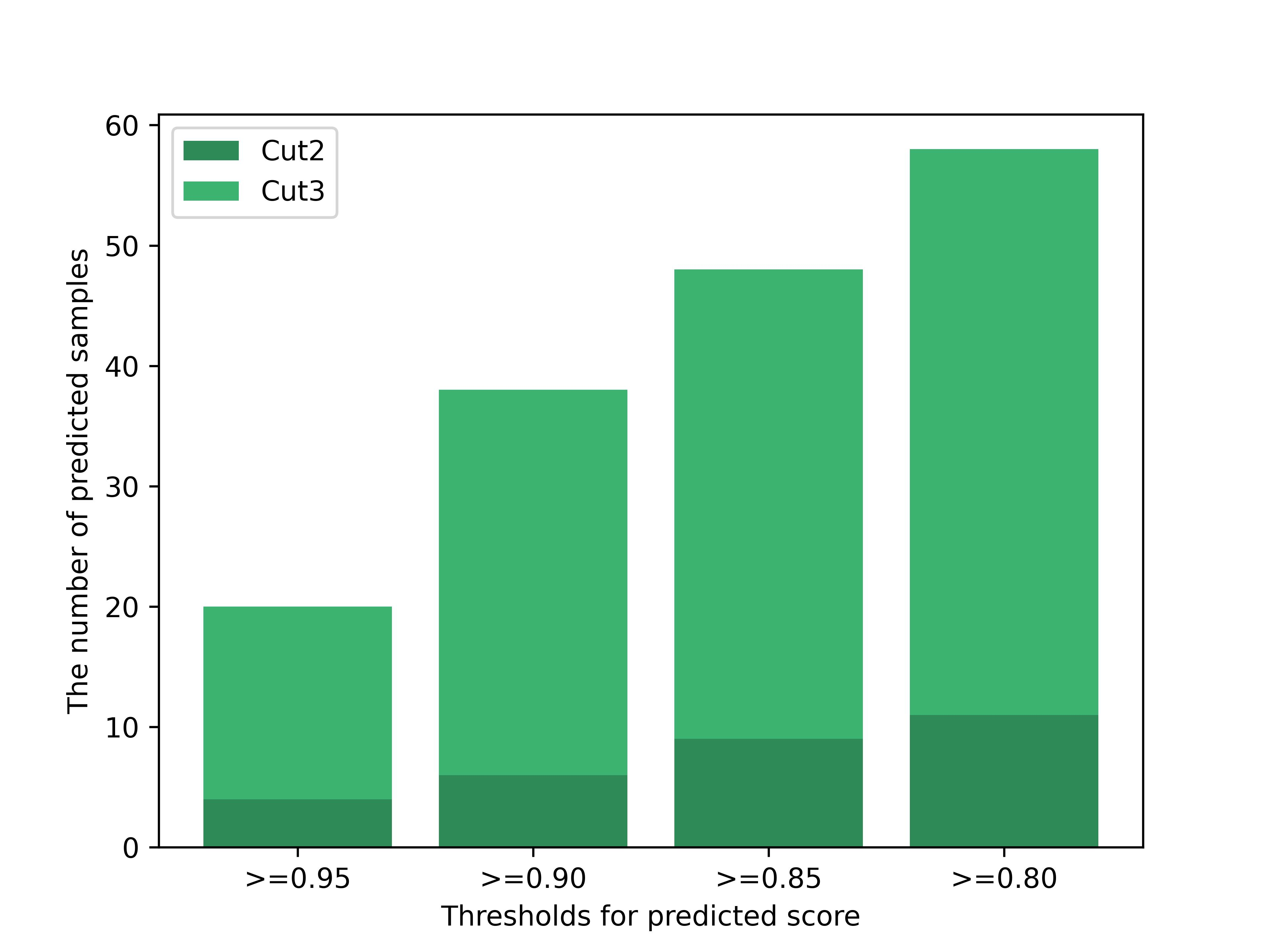

Supplement: S1 Fig — (TIF) [file pone.0309132.s005.tif]
